# Comparative analysis of sports consumption habits in Hungary, Poland and Germany

**DOI:** 10.1186/s12889-020-09442-6

**Published:** 2021-04-23

**Authors:** Dávid Paár, Antal Kovács, Miklós Stocker, Márk Hoffbauer, Attila Fazekas, József Betlehem, Barbara Bergier, Pongrác Ács

**Affiliations:** 1 University of Pecs, Faculty of Health Sciences, Pecs, Hungary; 2 Corvinus University of Budapest, Corvinus Business School, Budapest, Hungary; 3 Pope John Paul II State School of Higher Education in Biala Podlaska, Biala Podlaska, Poland

**Keywords:** Sports consumption, Sports venues, household’s expenditures, Sports services, Sports shoes, Sportswear

## Abstract

**Background:**

The so-called sports consumption models are looking for the factors that influence the sports spending of households. This paper aims to examine the Hungarian, Polish and German households’ sports expenditures which can be an important indicator of physical activity and sporty lifestyle.

**Methods:**

Surveying of households in three countries (Hungary, Poland and Germany) has been conducted with a self-designed questionnaire. We have used descriptive and bivariate non-parametric and parametric statistical methods: (1) χ^2^ test, Mann-Whitney test and Kruskal-Wallis test for checking the relationship between sociodemographic and physical activity variables and (2) independent sample t-test and ANOVA for checking the differences in sports expenditures.

**Results:**

Our research concluded that men, especially previous athletes, exercise more than women and those who have no history as registered athletes. The choice of sports venues is obviously different between the countries in the sample. Members of the study population spend the most on sports services while they spend the least on sports equipment. German households have the highest spending rates compared to the other two countries.

**Conclusions:**

Results are in line with our previous research findings and with other literatures. The difference in preferences of sports venues could have the reason of different supply of sports clubs or the different living standards too. It needs further researches to clear it. Material wealth, income level and sport socialisation can be a determining factor regarding the level of sports spending.

## Background

The end consumers and the consumer sports market mean the heart of the sports industry, which fundamentally determine the development and growth potential of further sports markets (e.g. the market of sponsorship, sports equipment, sportswear, sports professionals, etc.) for leisure sports and professional sports alike [1, 2].

The spending of sports consumers in the leisure and professional sports markets can be compared in different extent to the frequency and duration of sports activities that also require physical activity. The literature usually analyses people’s time spent in sports and their sports habits, as well as their expenditure on sports – which include both passive and active sports consumption –, using the same demographic, socioeconomic and sports socialization factors [3–7].

The study of active sports activities is not only constantly at the forefront of researches because the costs associated with it are generating an ever-growing market and the sports industry is becoming increasingly important in the developed, welfare states’ economies [8, 9]. It is also important to analyse them because there are a number of other health and economic benefits realised at the societal level in addition to pure market transactions. However these are not immediately realised and possibly not only by sports consumers in many cases but also by other members of society, by social welfare system or by enterprises that employ physically active people [10, 11].

As an extension of our previous research in Hungary [12–15] we have analysed and compared sports consumption of three EU member states (Hungary, Germany, Poland) in this study. Although there are several approaches of analysing sports habits [16], current questionnaire is based on the leisure-demand model elaborated in detail by Pawlowski [17].

We were interested in the influence of those sociodemographic and economic factors on sport consumption expenditures which are widely accepted in the literature as affecting the frequency and extent of participation in sports consumption. However these factors have been less frequently examined regarding the expenditure side. We have investigated whether there are any differences in sports spending between the three countries surveyed.

It is important for the stakeholders appearing on the supply side of the sports market to be aware of the differences between types of households consuming sports. Furthermore these results may determine governmental sports policy, as different interventions may be appropriate for stimulating active or passive sports consumption for the three countries.

## Methods

Sampling was done online using convenience sampling techniques. The online questionnaire was made using Google Docs™. The survey was carried out from January 2018 to October 2018 in the three countries (Hungary, Poland and Germany).

We used our own, anonymous, online self-administered questionnaire to collect the data which included questions about the sports habits and sports spending of households. The basic questionnaire was developed in 2011 (we had already used it two times in Hungary) and contained 45 closed questions that focused on sports consumption, healthy lifestyle and quality of life in addition to demographic factors. The data on sports consumption have been always gathered in the currency of the single countries and these values were converted at the current exchange rate (on the 21 January 2019, the euro average rate [1 EUR = 318.20 HUF]).

Microsoft Office Excel 2013 Standard suite and the SPSS Statistics 22.0 statistical package were used to analyse the questionnaire. We have used descriptive and bivariate statistical methods in statistical data processing. Mean values, dispersion and ratios were calculated as descriptive statistical indicators. Statistical tables and figures were prepared for presenting the data. Both parametric and non-parametric tests were used after checking normality tests as bivariate statistics. We have applied χ^2^ test, Mann-Whitney test and Kruskal-Wallis test when checking the relationship between the frequency categories of physical activity and some nominal and ordinal categories of sociodemographic variables and when checking the differences between countries’ preferred sports locations. Independent sample t-test has been used when checking the differences between men’s and women’s sports expenditures. ANOVA has been used to test the differences of sports expenditures by marital status, by previous personal sport history and by countries. The significance value was determined to be *p* < 0.05.

## Results

The number of interviewees was 566, of whom 68.6% were Hungarian (388), 19.4% Polish (110) and 12% German (68) citizens. The proportion of men (49.8%) and women (50.2%) was almost the same in the sample. The average age of respondents was 33.75 ± 12.4 years, the youngest participant was 18 years old and the oldest was 72 years old (Table 1). The respondents typically live in villages (17.3%), in towns with a population of 50,000 to 100,000 (15.9%), in cities with a population of 100,000 to 200,000 (14.8%) and in the capitals (7.2%). The respondents live in three (25.5%) or four (24.5%) member households most commonly.

**Table 1:** Summary of sample characteristics

Sample characteristics (***N*** = 566)		
**Age**		33.75 ± 12.4 ys
**Gender**	Male	49.80%
	Female	50.20%
**Level of Education**	Elementary	5.80%
	Vocational	9.50%
	Secondary	38.50%
	Tertiary	46.10%
**Marital status**	Single	41.50%
	Married	29.90%
	Divorced	6.90%
	Widow/er	1.10%
	Domestic Partnership	20.70%
**Nationality**	Hungarian	68.6%
	German	12%
	Polish	19.40%
**Total (respondents)**		566

Respondents mostly do sports alone (38%) or with friends (27.7%). They started to do sports primarily motivated by parents and friends (23.1%) or due to the popularity of the sport (17.7%). Only 11.5% of the respondents stated that they never have done sports in their childhood, while 63.3% reported practicing sport for more than 5 years in their childhood.

We have examined sociodemographic factors that have an effect on physical activity rates most frequently (Table 2).

**Table 2:** Summary of variables affecting physical activity rates

	Variables	Pearson χ^**2**^ value	Significance ***p*** value	Cramer V-value
**Physical activity variables**	Gender	23.44	0.000***	0.21
	Number of children	21.59	0.160	0.09
	Education	10.26	0.110	0.12
	Size of household	16.852	0.660	0.08
	Registered sports activity in the past	98.67	0.000***	0.24

*** *p* < 0.01

Cross-tabulation analysis shows after checking the standardised residuals that category doing physical activity regular (based on Eurobarometer nomenclature 1–2 or 3–4 times a week) typically includes men; moreover men who used to be registered athletes. The results of previous studies [15, 18] also draw a clear link between earlier habitual physical activity and current sports habits. Retired or currently active athletes are more likely to engage in regular physical activity. Women prefer non-traditional sporting forms, additionally they prefer to exercise at home individually or in groups in fitness clubs.

**Fig. 1:**
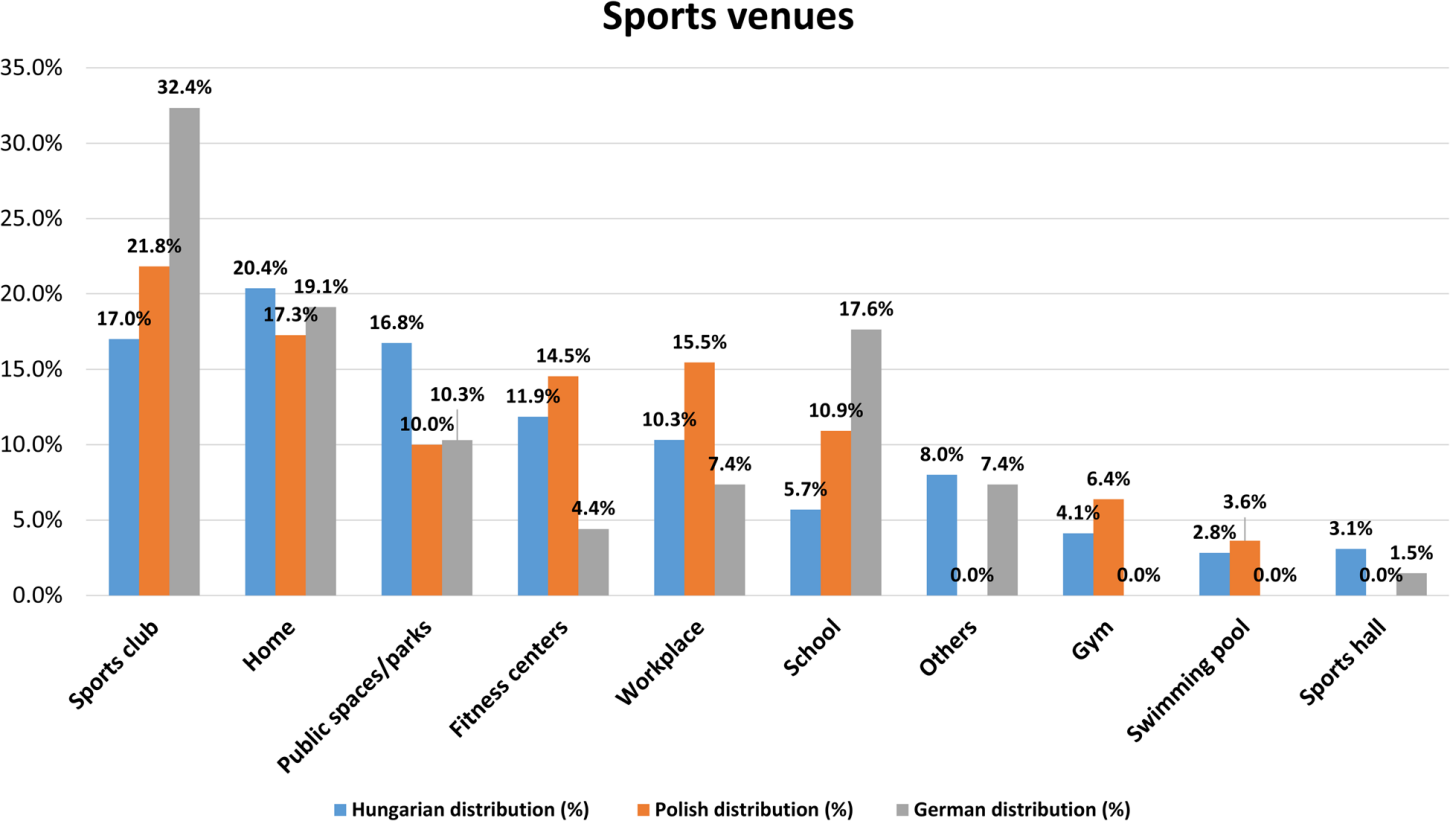
Preferred sports venues reported by the respondents of the three countries

We also have looked to establish a pattern regarding the choice of sports venues (Fig. 1). In terms of the countries surveyed, it can be stated that the respondents are the most active in sports clubs, at home and in public/outdoors/parks. We also have quantified the difference between the three countries in the ranking of preferred sports locations (χ^2^ = 48.645; *p* < 0.01) [15, 19–21].

It can be stated that most of the respondents prefer sports clubs in Poland and Germany, while the Hungarians prefer their home as a sports venue. Doing sports in the open air (public spaces and parks) is very common (16.8%) in Hungary, while this is less popular in Poland (10%) and Germany (10.3%).

We have examined the items and volume of annual sports expenditures.

**Fig. 2:**
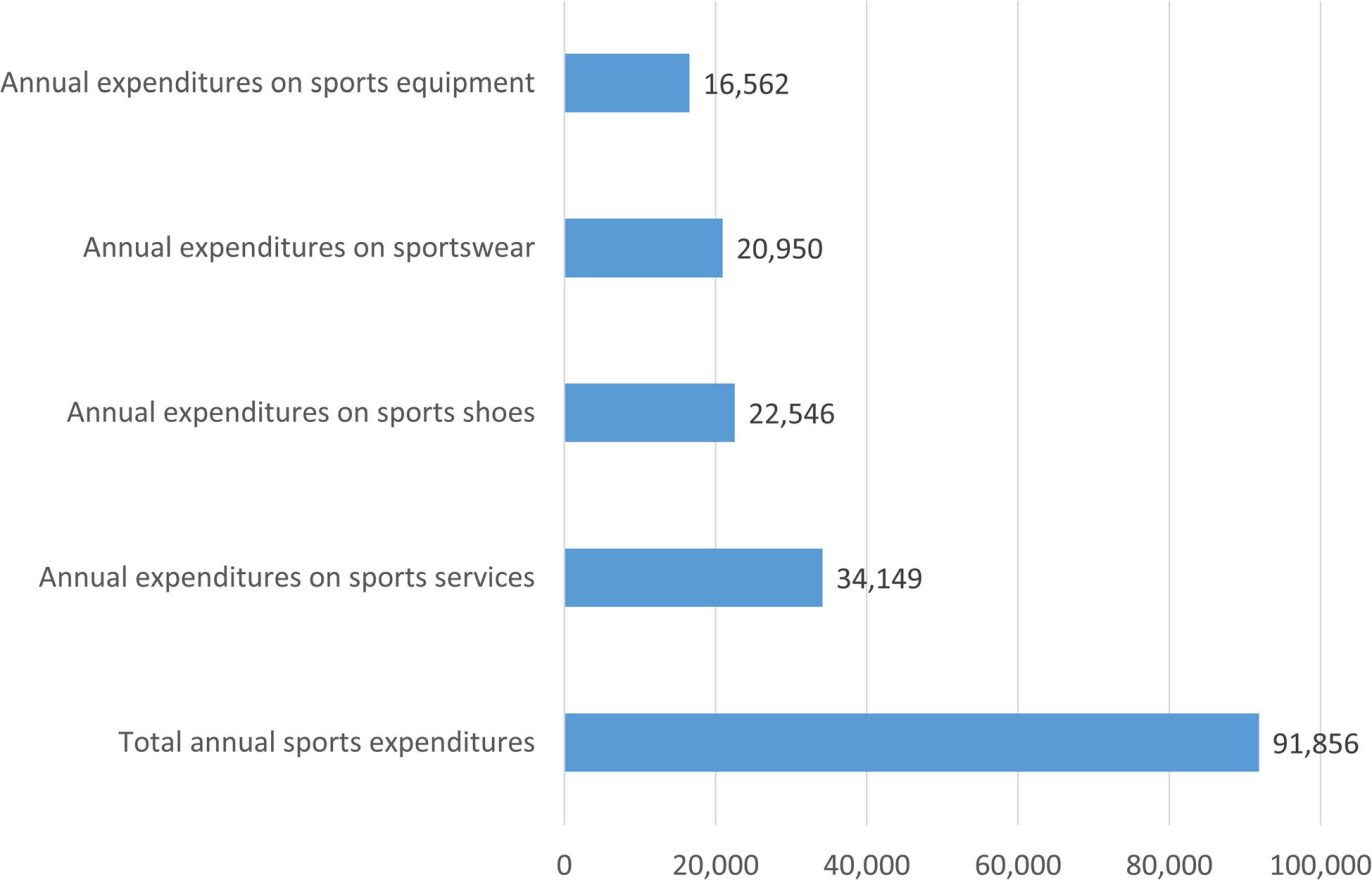
The aggregated values for sports expenditures per household for the whole sample in 2017 (HUF)

Figure 2 shows that the average value of annual sports spending is HUF 91,856 (EUR 288). It can be seen that the expenditure on sports services has the highest average value with HUF 34,149 (EUR 107) in the examined countries. This item is followed by sports shoes with a value of HUF 22,546 (EUR 70) and by sportswear with almost the same value of HUF 20,950 (EUR 66). It can also be stated that respondents spend the least amount of money on sports equipment with HUF 16,552 (EUR 52) on an annual basis.

We have examined the effects of sociodemographic and further factors on the volume of sports expenditure per item (Table 3).

**Table 3:** The average annual sports expenditure per household by gender of the household’s head in HUF (EUR)

	Gender					
	Female		Male		T-value	Significance ***p*** value
	Mean	Std. Deviation	Mean	Std. Deviation		
**Expenditure on sportswear**	19,540.44 (61)	30,093.15 (95)	22,370.37 (70)	35,514.43 (112)	1.01	0.317
**Expenditure on sports shoes**	22,769.51 (72)	30,840.93 (97)	22,323.42 (70)	31,762.46 (100)	−0.17	0.869
**Expenditure on sports equipment**	14,131.27 (44)	31,089.99 (98)	18,928.57 (59)	39,735.90 (125)	1.54	0.123
**Expenditure on sports services**	29,923.95 (94)	52,674.59 (166)	38,327.07 (120)	62,119.20 (195)	1.68	0.091
**Annual total expenditure**	83,527.27 (262)	93,416.08 (294)	100,275.73 (315)	118,510.19 (372)	1.84	0.061

It can be stated that men’s average spending is more in each examined category – with the exception of sports shoes – but these are not significant differences. Only the average annual total sports expenditure category shows a close valuee to the significance threshold (*p* = 0.061), and the difference in spending on sports services was significant at *p* < 0.01.

We have examined the annual sports spending and expenditure items in terms of education but no significant differences were found regarding the single items according to the one-factor variance analysis (ANOVA).

We have examined next the relationship between marital status and annual sports spending (Table 4).

**Table 4:** Sports spending per household by marital status in HUF (EUR in brackets)

		Sample size (no of people)	Mean	Std. deviation	F-value	Significance ***p*** value
**Average spending on sportswear**	Single	224	24,151.79 (76)	36,811.78 (116)	1.161	0.327
	Domestic partnership	113	17,920.35 (56)	27,764.36 (87)		
	Married	162	19,722.22 (62)	30,858.57 (97)		
	Divorced	37	18,513.51 (58)	32,826.67 (103)		
	Widow/er	6	6666.67 (21)	5163.98 (16)		
	In total	542	20,950.18 (66)	32,905.67 (103)		
**Average spending on sports shoes**	Single	222	27,319.82 (86)	38,411.93 (121)	4.181	0.002***
	Domestic partnership	112	25,446.43 (80)	34,579.54 (109)		
	Married	163	16,319.02 (51)	16,655.81 (52)		
	Divorced	35	14,714.29 (46)	12,657.41 (40)		
	Widow/er	6	6666.67 (21)	5163.98 (16)		
	In total	538	22,546.47 (71)	31,276.72 (98)		
**Average spending on sports equipment**	Single	219	19,703.20 (62)	39,550.55 (124)	1.246	0.290
	Domestic partnership	110	15,045.45 (47)	34,538.21 (109)		
	Married	157	15,636.94 (49)	34,994.17 (110)		
	Divorced	33	7878.79 (25)	8480.70 (27)		
	Widow/er	6	1666.67 (5)	4082.48 (13)		
	In total	525	16,561.90 (52)	35,779.73 (112)		
**Average spending on sports services**	Single	221	35,113.12 (110)	57,667.63 (181)	0.224	0.925
	Domestic partnership	109	33,899.08 (107)	56,770.16 (178)		
	Married	159	35,031.45 (110)	59,684.02 (188)		
	Divorced	34	26,176.47 (82)	53,542.09 (168)		
	Widow/er	6	25,000.00 (79)	61,237.24 (192)		
	In total	529	34,149.34 (107)	57,716.44 (181)		
**Total annual sports expenditure**	Single	225	104,666.67 (329)	122,642.96 (385)	2.78	0.005***
	Domestic partnership	113	90,486.73 (284)	101,014.86 (317)		
	Married	165	84,121.21 (264)	89,704.72 (282)		
	Divorced	38	61,842.11 (194)	87,914.43 (276)		
	Widow/er	6	40,000.00 (126)	63,874.88 (201)		
	In total	547	91,855.58 (289)	106,866.30 (336)		

*** *p* < 0.000

The study shows that significant differences were found in the annual average sports spending category (*p* = 0.005) and in the annual average sports shoes expenditure category (*p* = 0.002). Singles spend significantly more compared to widow/ers and divorcees in both categories based on our results.

We have got an interesting result when we checked the annual sports spending categories by the factor registered athlete history (Table 5). The three categories (I have never been a registered athlete; Yes, but I am retired; I am still a registered athlete) differed significantly in each spending categories (*p* < 0.000). It can be seen that spending by currently registered athletes is always the highest in each categories while spending is the lowest in the case of people without history of being registered athletes. We only identified one exception in the case of sports services, as respondents who have never been registered athletes are ranked the second in spending on sports services.

**Table 5:** Correlation between sports spending and competitive sport in HUF (EUR)

Sports spending	Past registered athlete	Mean	Std. Deviation	Count	F value	Significance ***p*** value
**Expenditure on sportswear**	None	14,624.41 (46)	21,193.18 (67)	224	13.133	0.000***
	Yes, but retired	20,175.00 (63)	30,945.64 (97)	207		
	Yes, still active	33,134.92 (104)	46,672.19 (147)	132		
**Expenditure on sports shoes**	None	17,047.62 (54)	21,039.89 (66)	224	27.265	0.000***
	Yes, but retired	17,688.44 (56)	18,827.59 (59)	207		
	Yes, still active	39,841.27 (125)	50,015.74 (157)	132		
**Expenditure on sports equipment**	none	11,280.79 (35)	28,758.73 (90)	224	10.539	0.000***
	Yes, but retired	14,252.58 (45)	30,079.05 (95)	207		
	Yes, still active	29,040.00 (91)	49,382.05 (155)	132		
**Expenditure on sports services**	None	31,352.66 (99)	55,363.81 (174)	224	3.741	0.024**
	Yes, but retired	29,179.49 (92)	50,735.25 (159)	207		
	Yes, still active	46,250.00 (145)	69,125.27 (217)	132		
**Total annual sports expenditure**	None	71,976.74 (226)	88,133.15 (277)	224	48.349	0.000***
	Yes, but retired	79,257.43 (249)	87,791.83 (276)	207		
	Yes, still active	146,141.73 (459)	141,187.13 (444)	132		

** *p* < 0.05; *** *p* < 0.01

The relative value and structure of annual sports spending have been examined too (Tables 6 and 7). Variance analysis showed that we can identify a significant difference between the researched countries (F = 6.427; *p* = 0.002) regarding the average annual total sports expenditure (HUF 91,885).

**Table 6:** Indicator of annual sports spending per household in HUF (EUR in brackets) in 2017

Nationality	Annual sports expenditures (2017)				
	Mean	Std. Deviation	Sample size (no. of people)	F value	Significance ***p*** value
**Hungarian**	89,986.45 (283)	102,981.04 (324)	388	6.427	0.002***
**Polish**	73,681.82 (232)	96,173.77 (302)	110		
**German**	131,397.06 (413)	132,751.72 (417)	68		
**In total**	**91,855.58 (289)**	**106,866.30 (336)**	**566**		

*** *p* < 0.000

**Table 7:** Annual sports spending per household per item in HUF (EUR) in the surveyed countries in 2017

Sports expenditure items	Nationality	N	Mean	Std. Deviation	F value	Significance ***p*** value
**Expenditure on sportswear**	Hungarian	364	19,505.49 (61)	31,915.91 (100)	2.062	0.128
	Polish	110	21,181.82 (67)	30,732.10 (97)		
	German	68	28,308.82 (89)	40,266.00 (127)		
**Expenditure on sports shoes**	Hungarian	360	22,277.78 (70)	32,546.61 (102)	0.117	0.889
	Polish	110	22,363.64 (70)	24,200.13 (76)		
	German	68	24,264.71 (76)	34,762.14 (109)		
**Expenditure on sports equipment**	Hungarian	347	15,792.51 (50)	34,434.20 (108)	1.705	0.183
	Polish	110	14,454.55 (45)	33,306.65 (105)		
	German	68	23,897.06 (75)	44,936.49 (141)		
**Expenditure on sports services**	Hungarian	351	35,911.68 (113)	60,041.85 (189)	10.57	0.000***
	Polish	110	15,681.82 (49)	28,344.84 (89)		
	German	68	54,926.47 (173)	71,401.18 (224)		
**Total annual sports expenditure**	Hungarian	369	89,986.45 (283)	102,981.04 (324)	6.427	0.002***
	Polish	110	73,681.82 (232)	96,173.77 (302)		
	German	68	131,397.06 (413)	132,751.72 (417)		

*** *p* < 0.000

The results supported our hypothesis that the average annual sports spending of German households is the highest HUF 131,397 (EUR 413), followed by Hungarian ones.

It seems that there is a significant difference between the examined nations only in terms of expenditures on sports services and in the total annual sports expenditure. It is clear that the sports consumption of German households is the highest for both items.

## Discussion

Our research concluded that men – especially previous athletes – exercise physical activity more than women and those who have no history as registered athletes. This result is in line with our previous research findings [14, 15]. The international literature’s similar results suggest that men do sports with higher possibility than women and they send more time with it too. However there are some sports with different characteristics – these are the so called sports for women [22–25]. Based on Breuer and Wicker [26] the gender differences decrease with age.

The choice of sports venues is obviously different between the countries in the sample. Doing sports at home is preferred the most in Hungary – which is in line with our previous results [14, 15] – sports clubs are dominantly the venue of choice in the other two countries at the same time. The question arises whether the reason for this is the limited choice possibilities of sports clubs or the lack of available income that limits Hungarian consumers from using such services.

Members of the study population spend the most on sports services, while spending the least on sports equipment as it was expected on the basis of previous researches conducted in Hungary [12, 14, 15]. Although there is no significant difference between amount of sports expenditures of single items, it can be clearly stated that men spend more overall on sports than women, which is also consistent with findings by previous researches. Similarly, it has been confirmed that singles spend the most on sports.

Although there is an identifiable trend that the increase in the level of education results increase in sports spending but it is not significant. One of the reasons could be that the sample size at some levels of education was relatively low. There is some contradiction between our conclusions and some international researches that suggest that more educated social groups have increased willingness for engaging in sports [3, 4, 27, 28].

Not surprisingly, German households with the highest living standards have the highest spending rates compared to the other two countries. This aspect is only significant in the category of annual total sports expenditures and annual sports service expenditures – Polish and Hungarian spending figures do not differ significantly in the case of sportswear, sports shoes and sports equipment. However, a similar trend is observable in all categories when comparing category averages which suggests that material wealth and income level can be a determining factor in the amount of sports spending [13].

Women prefer non-traditional sports opportunities based on the results of our study which is consistent with international researches [19]. They prefer to exercise at home individually or in groups in fitness clubs. Breuer et al. [29] identified cycling and running as men dominated sports, however women were overrepresented in swimming, fitness and gymnastics. Borgers et al. [30] identified cycling as a sport which is done with higher frequency by men and men spend more time on a cycling, running or tennis training session like women.

It is worth comparing our results with Eurobarometer [31, 32] data. While the citizens of the EU would like to do sports in parks and in nature, Hungarian citizens would like to do the same mostly at home. Some locations popularity grew during the past period in Hungary. The popularity of doing sports at home (8%), in the workplace (6%), in a sports centre (3%) or in fitness-wellness centres (3%) continued to increase comparing the figures of Eurobarometer 2014 and 2018 [31, 32]. There is a significant difference in terms of the popularity of sports centres, sports clubs and fitness-wellness centres, as these sports venues are much less popular in Hungary compared to the EU. The most popular sports venues are parks in 17 of the countries in the EU, it is most prominently in Finland (67%). Respondents nominated the household as the most popular sports venue in nine countries, mainly in Eastern Europe.

The popularity of parks and open-air venues decreased slightly in Germany but they still remained the most popular sports venues in 2018 (39%) consistent with the average EU rate (40%). Home is ranked as the second most popular sports venue both in Germany and the EU in 2018, similarly to the 2014 figures (46%), although this popularity decreased slightly (41%)., The popularity of doing sports in the workplace (18%) and at sports centres (9%) continued to increase compared to 2014 data of Germany. The popularity of fitness centres decreased slightly (14%) in 2018; however, the popularity of sports clubs (21%) and universities (4%) did not change.

More than half of the respondents (53%) believe that the authorities do not make a sufficient effort at local level to provide residents appropriate infrastructure to do physical activity in Poland. Polish positive respondents identified parks and outdoor venues as the most frequent sports venues (42%). This location was second placed (35%) in 2014 and 37% of respondents marked their household as the sports venue they chosen in that year., The number of people exercising in health and fitness centres and sports clubs did not change significantly in 2018 compared to the data gathered 4 years previously. However more than twice as many people (15%) exercised in sports centres in 2018 then in 2014 (6%) [31, 32].

### Limitations

It must be stated that the sample cannot be considered as representative one because of the convenience sampling techniques. However it is able to show tendencies due to large sample size.

Our research is limited only for three countries however a wider range of the EU states would be interesting regarding their households’ sport expenditures similarly like researches of Downward et al. [20] and Hovemann and Wicker [33] about sports participation rates in the EU.

## Conclusion

The main findings of the paper are that men – and especially men with previously registered sport history – are more involved into physical activity like women. However we didn’t find a significant effect of the children number in the households, respondents’ educational level and size of households.

There are big differences in preferred sports venues of the three countries. Sports clubs are extremely popular in Germany whilst Hungarians prefer home and public park activities.

The most important sports expenditure category is sports services and we can find significant differences regarding the category total sports expenditures based on the marital status of the respondents. Previous individual sports past supports the amount of sports expenditures in all categories and German households spend significantly more on sports than Hungarians and Poles.

## Data Availability

The datasets used and/or analysed in the current study are available from the corresponding author on reasonable request.
